# A Fast, Low‐Temperature Synthesis Method for Hexagonal YMnO_3_: Kinetics, Purity, Size and Shape as Studied by In Situ X‐ray Diffraction

**DOI:** 10.1002/chem.202000528

**Published:** 2020-05-12

**Authors:** Kenneth P. Marshall, Anders B. Blichfeld, Susanne L. Skjærvø, Ola G. Grendal, Wouter van Beek, Sverre M. Selbach, Tor Grande, Mari‐Ann Einarsrud

**Affiliations:** ^1^ Department of Materials Science and Engineering NTNU—Norwegian University of Science and Technology Sem Sælands vei 12 7034 Trondheim Norway; ^2^ Swiss-Norwegian Beamlines European Synchrotron Radiation Facility 71 avenue des Martyrs, CS 40220 38043 Grenoble Cedex 9 France

**Keywords:** hydrothermal synthesis, in situ, synchrotron, YMnO_3_

## Abstract

The reaction mechanisms, phase development and kinetics of the hydrothermal synthesis of hexagonal‐YMnO_3_ from Y_2_O_3_ and Mn_2_O_3_ using in situ X‐ray diffraction are reported under different reaction conditions with temperatures ranging from 300 to 350 °C, and using 1, 5 and 10 m KOH, and 5 m NaOH mineraliser. Reactions initiated with Y_2_O_3_ hydrating to Y(OH)_3_, which then dehydrated to YO(OH). Higher temperatures and KOH concentrations led to faster, more complete dehydrations. However, 1 m KOH led to YO(OH) forming concurrently with Y(OH)_3_ before Y(OH)_3_ fully dehydrated but yielded a very low phase purity of hexagonal‐YMnO_3_. Using NaOH mineraliser, no YO(OH) was observed. Dehydration also initiated at a higher temperature in the absence of Mn_2_O_3_. The evolution of Rietveld refined scale factors was used to determine kinetic information and approximate activation energies for the reaction. The described hydrothermal synthesis offers a fast, low‐temperature method for producing anisometric h‐YMnO_3_ particles.

## Graphical abstract

**Phase development**: The hydrothermal synthesis of hexagonal YMnO_3_ from Y_2_O_3_ and Mn_2_O_3_ was investigated by in situ X‐ray diffraction. Using this technique, information on mechanism, kinetics and thermodynamics of the reaction has been extracted, as well as the impact that mineraliser type and concentration, and temperature have on these properties.

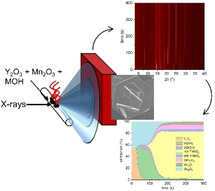

## Introduction

Hexagonal YMnO_3_ (h‐YMnO_3_) is a multiferroic material with simultaneous ferroelectric and antiferromagnetic properties.^1^, ^2^, ^3^, ^4^, ^5^, ^6^, ^7^ These properties have led to a large research interest due to the possibility for ferroelectric‐antiferromagnetic coupling,^8^ allowing for potential applications in, for example, antiferromagnetic memory devices.^9^ In addition to their ferroic properties, hexagonal manganites also display oxygen storage capacity,^10^ which may be useful as oxygen membranes in gas separation or hydrogen fuel cell applications. h‐YMnO_3_ has also been reported to display photocatalytic properties,^11^ and for this application, small particles with high surface area are most appropriate.

h‐YMnO_3_ crystallises in the space group *P*6_3_ *cm* at room temperature.^12^ There exists an orthorhombic perovskite phase of YMnO_3_ (o‐YMnO_3_) which is kinetically stable at room temperature, and is the thermodynamically stable phase at high pressure due to its higher density compared with h‐YMnO_3_.^13^, ^14^ At room temperature, the most stable phase in the Y‐Mn‐O system is YMn_2_O_5_, as determined by Chen et al.,^15^ who calculated the phase diagram for this system at atmospheric pressure in air. According to this work, h‐YMnO_3_ becomes the most thermodynamically stable phase above 789 °C.

Solid‐state and sol‐gel methods are well established to prepare highly pure h‐YMnO_3_. Both methods have the drawback of requiring at least one high temperature crystallisation step above 789 °C, where h‐YMnO_3_ becomes the most thermodynamically stable phase. The sol‐gel method has been used to prepare nanoparticles with control over the crystal and grain size based on the crystallisation temperatures in the range of 800–1100 °C, giving particle sizes of the order of 100 nm. However, in addition to the high temperature required, another drawback is the amount of time and number of steps required; amorphous precursors require drying from solution at around 150 °C, calcination at ≈500 °C, and crystallization to form h‐YMnO_3_.^12^, ^16^, ^17^ Additionally, our previous studies on the sol‐gel synthesis of h‐YMnO_3_ have highlighted the effect of oxidising and reducing conditions on the outcome of the reaction; oxidising conditions were shown to lead to a higher amount of orthorhombic phase, due to the smaller Mn^IV^ B‐site cation causing the perovskite structure to become more geometrically favourable.^10^, ^16^, ^18^

Hydrothermal synthesis, in contrast to other methods, typically requires only one step, is performed at lower temperatures, and can give phase pure products from reactions lasting only a matter of hours. Still, challenges remain in synthesising h‐YMnO_3_ hydrothermally, especially the range of oxidation states that Mn is capable of adopting, since Mn is stable from +2 to +4, with +2 being the most stable state.^19^ Manganese is not stable in the +3 oxidation state in water, therefore the possibility for Mn^3+^ to disproportionate in solution adds a considerable challenge for the hydrothermal synthesis of YMnO_3_. Indeed, experimental evidence and theoretical calculations have shown that h‐YMnO_3_ is capable of accommodating significant oxygen interstitial defects, compensated by the presence of Mn^IV^, up to an oxygen stoichiometry of YMnO_3.14_.^20^, ^21^ This variable oxidation state gives rise to a stable secondary phase in the system, YMn_2_O_5_. o‐YMnO_3_ can also form as it has a Goldschmidt tolerance factor (*t*) of 0.80 and an octahedral factor (*μ*) of 0.51 (high spin), which is within the range in which oxide perovskites can form (*μ*>0.425, and 0.906–0.232*μ*<*t*<1).^22^, ^23^ In literature, there have been two reported pathways for synthesising h‐YMnO_3_ by hydrothermal routes. One method was reported by Stampler et al.^24^ in which hexagonal LnMnO_3_ (Ln=Ho–Lu, or Y) was synthesised from the oxides with KOH mineraliser (although results for Ln=Y were not included in the manuscript). The use of a high‐temperature autoclave was required for the larger rare earth metals due to the higher stability of Ln(OH)_3_. In an alternative method, aqueous Y(NO_3_)_3_, a Mn^II^ salt, and KMnO_4_ were reacted with KOH,^25^ or NaOH,^26^ at 240 °C. This latter route has been used to obtain particles with hexagonal^25^ and nanorod^26^ shapes. Under similar conditions to this route, Zhou et al. synthesised o‐YMnO_3_.^27^ The primary difference between the two routes appears to be that a lower concentration of KOH and a lower filling factor are required to form the hexagonal phase compared with the orthorhombic phase.

The work by Stampler et al.^24^ on the hydrothermal synthesis of hexagonal manganites highlighted the importance of temperature with regards to the choice of Ln atom, inferring that the Ln_2_O_3_ is converted to Ln(OH)_3_ which then dehydrates to LnO(OH). However, the reaction progression at different temperatures and what happens with the Mn_2_O_3_ during the reaction remain uncertain.

In situ X‐ray diffraction (XRD) during hydrothermal synthesis is a technique that has been used since the 1990s, with early papers having been published on zeolites by Norby et al.^28^, ^29^ Walton and O'Hare published an early review on the topic (for both hydrothermal and solid‐state reactions) in 2000.^30^ In situ neutron diffraction,^31^ energy dispersive XRD,^32^ and angle dispersive XRD^28^ have all been used to monitor the process of crystallisation by hydrothermal synthesis. In situ diffraction has been used to probe, among other systems, the formation under hydrothermal conditions of functional binary metal oxide nanoparticles,^33^, ^34^, ^35^ and several ferroic materials, including BaTiO_3_,^36^, ^37^, ^38^ K_1−*x*_Na_*x*_NbO_3_,^39^, ^40^ BiFeO_3_,^41^ and Sr_1−*x*_Ba_*x*_NbO_3_.^42^

Here we report in situ hydrothermal synthesis of h‐YMnO_3_ from Y_2_O_3_ and Mn_2_O_3_ at temperatures in the range of 300 to 350 °C monitored by synchrotron XRD. This study has allowed us to quantitatively measure the phases which appear over the course of the reaction, thereby revealing the reaction progression and kinetics, under different conditions. We have also studied the effect of hydrothermal conditions on Y_2_O_3_ to reveal the formation of the different hydroxides under increasing temperature. The results are discussed in relation to those obtained using ex situ techniques from reactions performed in a high‐temperature autoclave.

## Results and Discussion

### In situ XRD‐determined reaction progressions

**Figure 1:**
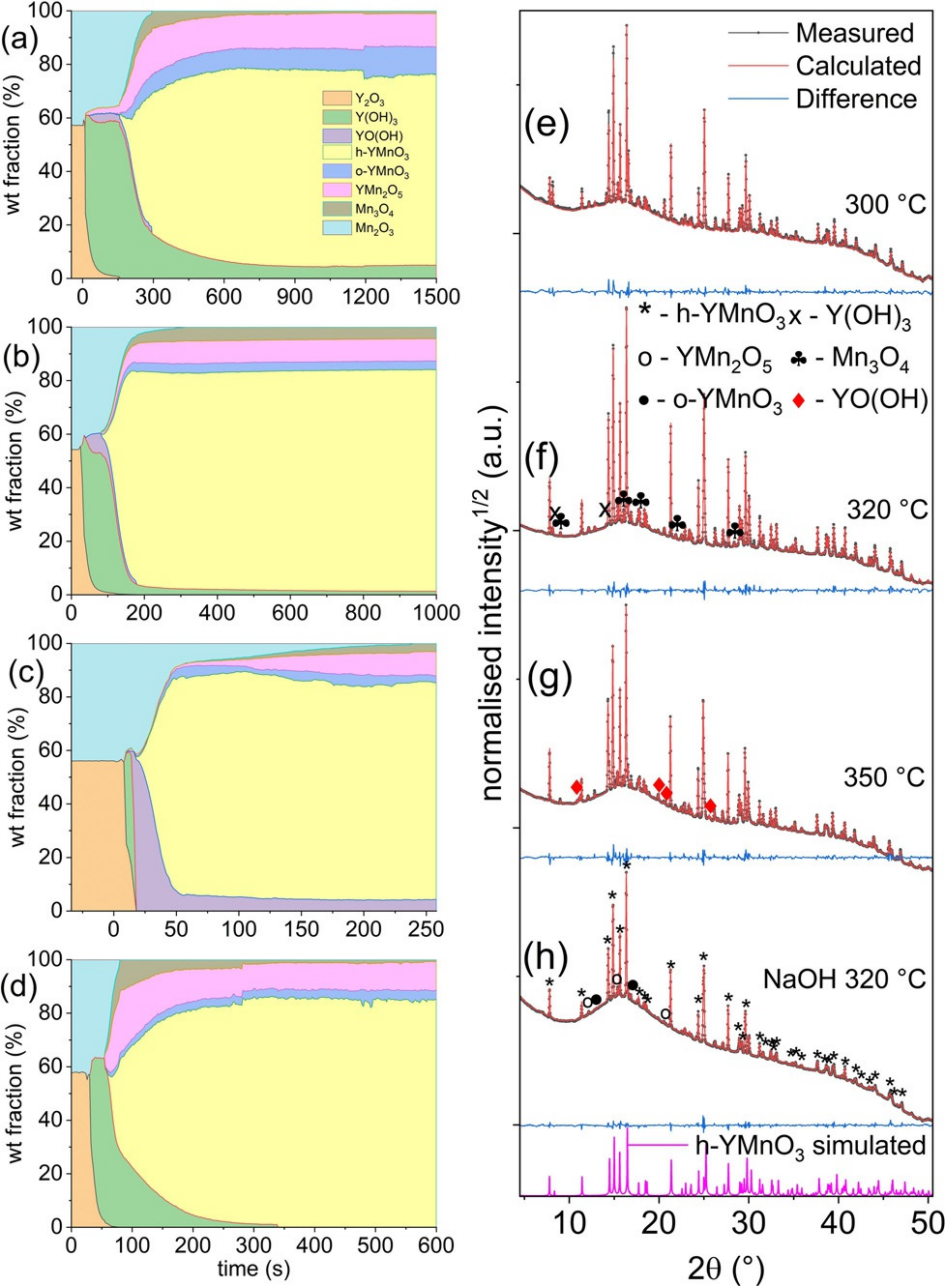
Stacked area plots of the weight fractions of observed phases over time for the reaction between Y_2_O_3_ and Mn_2_O_3_ in 5 m KOH at (a) 300 °C, (b) 320 °C, and (c) 350 °C, and (d) in 5 m NaOH at 320 °C. Integrated XRD patterns (*λ*=0.77445 Å) and patterns calculated using Rietveld refinement of the final dataset for the reaction at (e) 300 °C, (f) 320 °C, and (g) 350 °C, and (h) 320 °C in 5 m NaOH.

Figures 1 (a)–(d) show the weight fractions of observed phases over time for in situ reactions between Y_2_O_3_ and Mn_2_O_3_ in 5 m KOH at 300, 320 and 350 °C, and in 5 m NaOH at 320 °C monitored by synchrotron XRD. Integrated XRD patterns and patterns calculated using Rietveld refinement of the final dataset of the reactions are included in Figures 1 (e)–(g). The 2D plot of the XRD patterns over time, along with the refined patterns at the beginning, intermediate period and end of the reaction at 320 °C in 5 m KOH are shown in Figures S1 (a) and (b) in Supporting Information. The reactions show different progressions; the reactions at 300 and 320 °C progresses via an intermediate stage in which the Y_2_O_3_ converts mostly to Y(OH)_3_, and a small amount of YO(OH) which appears several seconds later before both are converted into h‐YMnO_3_ In addition, o‐YMnO_3_ and YMn_2_O_5_ are present, although some Y(OH)_3_ remains. At 350 °C, the Y(OH)_3_ is decomposed more easily and appears only briefly after the heat is applied before completely decomposing into YO(OH). The latter then reacts with Mn_2_O_3_ to form h‐YMnO_3_ and secondary phases, but also remaining in a significant quantity. This reaction progression (Y_2_O_3_ to Y(OH)_3_ to YO(OH)) is in line with previously published work.^24^, ^43^ The secondary phase YMn_2_O_5_, in which Mn has an average oxidation state of 3.5, is the major secondary phase at all three temperatures. YMn_2_O_5_ forms due to charge disproportionation of Mn, leading to the concurrent formation of Mn_3_O_4_, in which Mn has an average oxidation state of $2\frac{2}{3}$ . YMn_2_O_5_ being the most stable phase in the MnO_*x*_‐YO_1.5_ phase diagram below 789 °C explains why this charge disproportionation occurs.^15^ However, from weight fractions of crystalline phases alone, the oxidation states do not fully balance; for example, the average oxidation state of Mn in YMn_2_O_5_ and Mn_3_O_4_ with weight fractions of 8.5 and 2.8, respectively (as in the reaction at 320 °C in 5 m KOH) is 3.2. This average oxidation number may be accounted for by a significant error in refining weight fractions with such low intensity, or the presence of soluble Mn^II^ or amorphous species not included in the Rietveld refinements. Another challenge with the hydrothermal synthesis of h‐YMnO_3_ is the appearance of significant amounts of o‐YMnO_3_ (except in the case of 1 m KOH at 320 °C). The final weight fractions of all observed phases of reactions at 300, 320 and 350 °C are shown in Table S1, where it can be seen that the reaction at 320 °C had a slightly higher proportion of h‐YMnO_3_. Using 5 m NaOH mineraliser at 320 °C, no YO(OH) was observed (Figure 1 (d)). Weight fraction evolutions for experiments using 1 and 10 m KOH, as well as with a partial substitution of Mn_2_O_3_ with Mn_3_O_4_ (weight fraction) presented in Figure S2 show that the reaction performed in 1 m KOH yields very low purity, while higher KOH concentrations lead to faster reactions. Both 10 m KOH and 1 m KOH reactions led to complete dehydration of Y(OH)_3_, and the use of Mn_3_O_4_ in the reaction resulted in a lower weight fraction of YMn_2_O_5_, although Mn_3_O_4_ remains after the reaction.

The h‐YMnO_3_ was fitted using the Stephens model for anisotropic peak broadening.^44^ The evolution of the (4 0 0) and (0 0 4) Stephens parameters over time from the onset of h‐YMnO_3_ formation are shown in Figure S3. A higher Stephens parameter indicates broader peaks from planes perpendicular to the indicated crystal axis for example, a large (4 0 0) parameter means broad (*hk*0) peaks.

**Figure 2:**
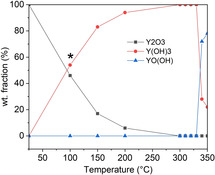
The evolution of weight fractions of different phases with increasing temperature as Y_2_O_3_ reacts in 5 m KOH solution. An unknown phase was also observed at 100 °C. The data shown are from diffraction patterns taken at equilibrium at each temperature. *An unidentified phase was observed at 100 °C, and this phase is not included in the calculations of the weight fractions.

The reaction of Y_2_O_3_ in 5 m KOH was followed under in situ hydrothermal conditions at temperatures in the range 100 to 350 °C. After stabilising at 100 °C, three phases were observed: unreacted Y_2_O_3_, Y(OH)_3_, and a third, unidentified phase. This third phase was not observed at 150 °C, where only Y(OH)_3_ and a small amount of Y_2_O_3_ appeared. In the range from 280 to 330 °C, Y(OH)_3_ was the only observed phase, and at 340 and 350 °C, YO(OH) appeared alongside Y(OH)_3_ (Figure 2, selected XRD patterns shown in Figure S4). After cooling to room temperature, the composition did not change from this.

### Kinetics

Using the scale factors extracted from the Rietveld refinement, information on the kinetics of h‐YMnO_3_ formation was analysed using the Johnson—Mehl—Avrami–Kolmogorov equation,^45^, ^46^ $\alpha =1\;-e^{kt^{n}}$ , where *α* is the extent of reaction. This equation was used to extract vales for *k*, a rate constant related to radial crystal growth, and *n*, an exponent related to the dimensionality of crystal growth, and is indicative of the growth and transport mechanisms^46^ (see Table 1). To choose appropriate values for the beginning of nucleation, the data were fitted such that the value for *n* was the same to 1 decimal place. An obtained value of *n*≥2 implies a reaction mechanism limited by nucleation and growth.^47^, ^48^ In this case, the evolution of α over time is characterised by an initial increase in rate, followed by an approximately constant rate, and a decrease in rate at the end of the reaction due to the depletion of reactants. Lower values of *n* indicate that diffusion or phase boundary growth can be limiting factors. There is one reaction where *n*<2; the reaction with 1 m KOH at 320 °C. There appears to be a relatively linear evolution (with respect to higher KOH concentrations) of the scale factor over time. Therefore, a precursor dissolution limiting mechanism could be possible in this reaction due to the low mineraliser concentration.

**Table 1:** Values for rate constant *k*, exponent *n*, and coefficient of determination calculated, R^2^, determined from the plots of normalised scale factors of h‐YMnO_3_ over reaction time using the Johnson–Mehl–Avrami–Kolmogorov model.

Temperature [°C]	[KOH] [m]	*K* [s^−1^]	*n*	*R* ^2^
320	1	1.8×10^−4^ (1)	1.4±0.01	0.974
300	5	1.1×10^−5^ (1)	2.4±0.03	0.732
320	5	7.9×10^−5^ (8)	2.4±0.03	0.979
320 (repeat)	5	1.4×10^−4^ (2)	2.4±0.04	0.976
350	5	8.1×10^−4^ (6)	2.4±0.03	0.998
300	10	3.7×10^−5^ (4)	2.4±0.03	0.989
320	10	4.2×10^−4^ (7)	2.4±0.06	0.987
350	10	3.9×10^−3^ (7)	2.4±0.09	0.981
320	5, NaOH	9.6×10^−4^ (21)	2.4±0.1	0.634

**Figure 3:**
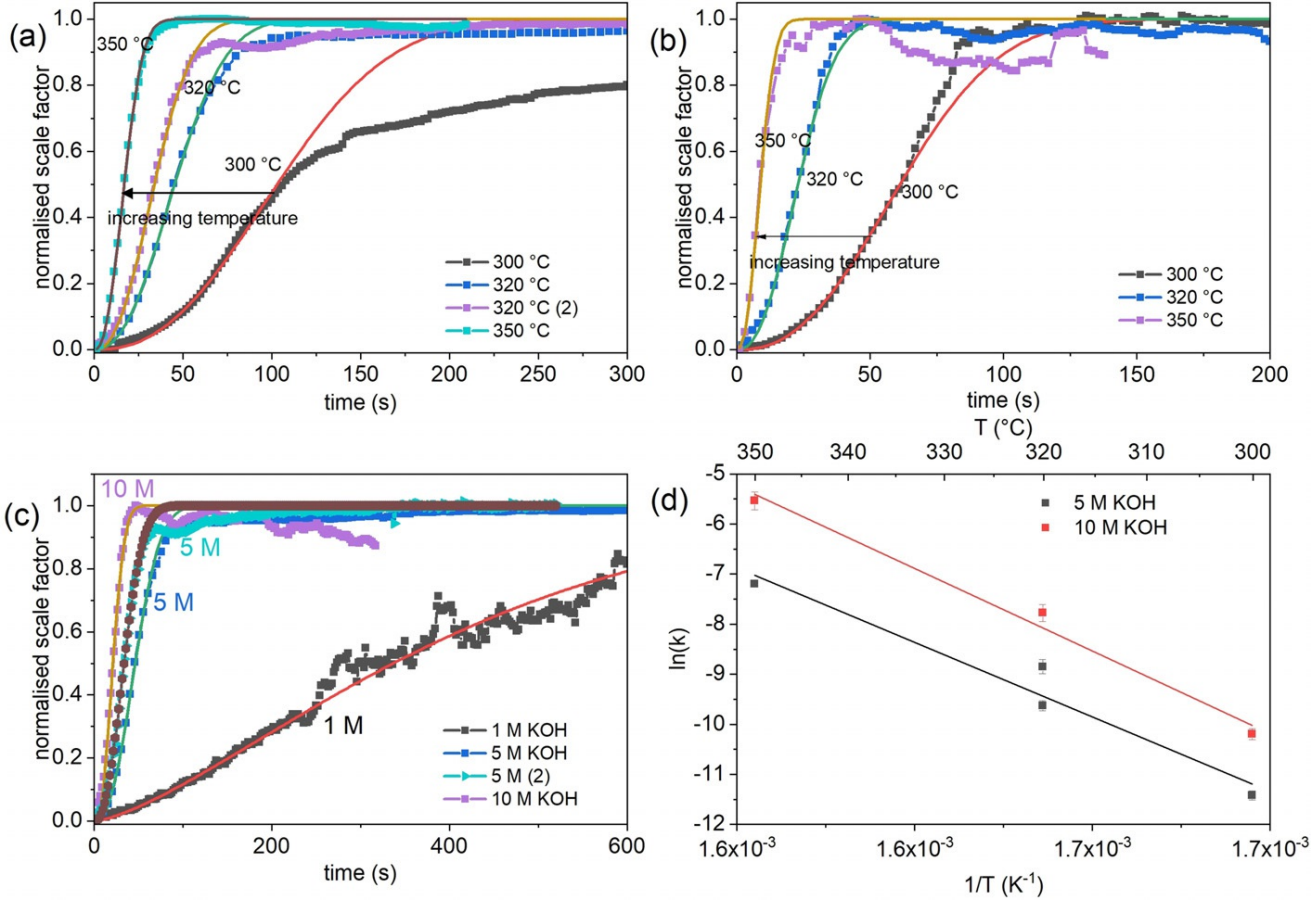
h‐YMnO_3_ scale factors and Avrami fits over time for reactions at 300, 320 and 350 °C in (a) 5 m KOH and (b) 10 m KOH, (c) evolution of h‐YMnO_3_ scale factors for reactions at 320 °C in 1, 5 and 10 m KOH, refined scale factors shown by line and scatter plots and fitted curves shown by solid lines, and (d) plot of ln(*k*) against 1/*T* for the corresponding reactions with linear regressions.

Figures 3 (a) and (b) show the evolution of scale factors over time for reactions at different temperatures at 5 and 10 m KOH, respectively. At 300 °C and 5 m KOH (Figures 1 (a) and 2 (a)) and at 320 °C in 5 m NaOH (Figures 1 (g) and S4), there is an abrupt reduction in the rate of h‐YMnO_3_ formation coinciding with the exhaustion of the Mn_2_O_3_. After this there is a small reduction in the weight fractions of Y(OH)_3_, Mn_3_O_4_ and YMn_2_O_5_ as h‐YMnO_3_ continues to form. This change in mechanism is the likely cause of the reduction in rate. Additionally, the four datasets at 320 °C and different KOH concentrations (with two reactions at 5 m) (Figure 3 (c)) show significantly different reaction rates, with higher KOH concentration increasing the reaction rate.

The progression of the quantity of h‐YMnO_3_ in reactions in 5 m KOH follows a definite trend according to the Arrhenius equation, with the linear regression giving an activation energy of 240±40 kJ mol^−1^. At 10 m KOH, a similar trend is observed with an activation energy of 270±30 kJ mol^−1^, but yielding higher *k* values for a given temperature (Figure 3 (d)). For comparison, hydrothermal processes which take place below 200 °C typically have activation energies below 100 kJ mol^−1^.^49^, ^50^, ^51^ The high stability of the intermediate phases (Figure 2) helps to explain the high temperature required and high activation energy for the reaction forming h‐YMnO_3_.

### Ex situ studies on autoclave synthesis

Reactions between Y_2_O_3_ and Mn_2_O_3_ to form h‐YMnO_3_ were also performed ex situ in an autoclave at 320 °C in KOH solution. Using a stoichiometric ratio of Y:Mn, a 90 % phase purity was achieved (measured by Rietveld refinement) after washing the product with acid to remove Y(OH)_3_. Using a 10 % excess of Y_2_O_3_, the phase purity was improved to 98 %, with minor secondary phases of YMn_2_O_5_ and Mn_3_O_4_ being observed. The XRD patterns show a large degree of preferred orientation in the (00*l*) direction, which is clearly indentifed from a simple comparison of the measured diffraction pattern and a simulated pattern of h‐YMnO_3_ without any orientation (Figure S6). The degree of orientation has been quantified by the March–Dollase model in diffraction pattern refinements (Table S2).

**Figure 4:**
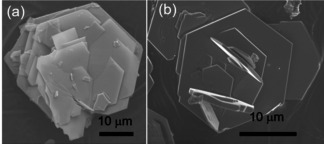
SEM images of h‐YMnO_3_ synthesised by a hydrothermal route, both (a) ex situ and (b) from the in situ capillary set‐up.

SEM images of the h‐YMnO_3_ formed both in situ and ex situ in the large autoclave showed similar large, flat hexagonal particles of the order of 10 μm across (Figure 4). This plate‐like structure explains the preferred orientation in the XRD patterns as the plates align during sample preparation.

### Discussion

**Figure 5:**
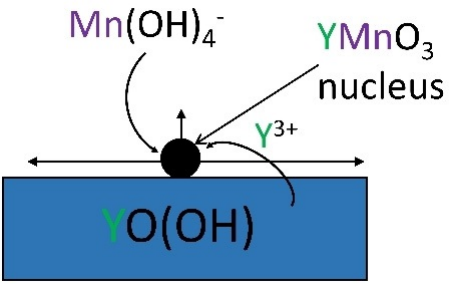
Schematic depicting the suggested mechanism of crystal growth of YMnO_3_ under hydrothermal synthesis.

The data presented here show the progression of the reaction between the two solid precursors Y_2_O_3_ and Mn_2_O_3_. The reaction proceeds via Y(OH)_3_ and/or YO(OH), while Mn_2_O_3_ disappears as the product appears, as shown by the weight fraction area plots in Figures 1 (a)–(d). The h‐YMnO_3_ appears concurrently with the reduction in scale factor of Mn_2_O_3_, Y(OH)_3_, and YO(OH). It is therefore likely that any soluble species would be extremely short lived, and that heterogeneous nucleation occurs on the surfaces of intermediate phases (Y(OH)_3_, YO(OH) or Mn_2_O_3_). It is also likely that a dissolution‐precipitation mechanism occurs rather than a shrinking‐core mechanism due to the fact that the YMnO_3_ forms in distinct hexagonal shapes which do not resemble the precursors. Stampler et al. suggested from ex situ studies that the reaction requires the breakdown of Ln(OH)_3_ into the more reactive LnO(OH) before proceeding to form h‐LnMnO_3_, while Mn_2_O_3_ is reactive at least as low as 150 °C. This was inferred from the fact that the required temperature for the reaction to proceed is heavily dependent on the stability of Ln(OH)_3_. The ionic radius dependence of Ln(OH)_3_ stability was reported by Klevtsov and Sheina.^43^ Stampler et al. then suggested that the LnO(OH) reacts with Mn(OH)_4_^−^ to form h‐YMnO_3_. Chouaib et al.^52^ and Kozawa et al.^53^ showed that Mn_2_O_3_ has a solubility of ≈2 mm in 5 m KOH solution at 25 °C. This may appear to be corroborated by our results which show the formation of YO(OH) in quantities dependent on reaction temperature before the appearance of h‐YMnO_3_ (Figure 1 (a)–(c)). However, this does not conclusively prove that h‐YMnO_3_ cannot crystallise directly from Y(OH)_3_ and Mn_2_O_3_, and in the reaction with 5 m NaOH at 320 °C, we do not observe any YO(OH) (Figure 1 (d)). A suggested mechanism for the formation of YMnO_3_ is depicted in Figure 5. For the aforementioned reasons, we consider it likely that nucleation occurs on the surface of one of the solid precursors. We also consider it likely that Mn‐species will have a higher solubility than Y‐species under the reaction conditions, assuming that Y will dissolve as a positive species, the equilibrium would shift towards solid YO(OH) or Y(OH)_3_ under a high OH^−^ concentration.

The data also show that the KOH concentration is an important factor for the reaction progression and rates. At higher KOH concentrations, the Y(OH)_3_ dehydrates into YO(OH) much faster; even at the low temperature of 300 °C in 10 m KOH, all of the Y(OH)_3_ eventually dehydrates (Figure S2 (b)). Related to this, we have also observed that when Y_2_O_3_ is heated in 5 m KOH solution, no dehydration of Y(OH)_3_ occurs until 340 °C. Taken together, this implies that soluble Mn is involved catalysing the dehydration of Y(OH)_3_, which we observe to take place at 300 °C in the presence of Mn_2_O_3_. It is also observed that using 1 m KOH leads to the concurrent formation of YO(OH) and Y(OH)_3_, before the Y(OH)_3_ fully dehydrates (Figure S2 (a)), which must result from a different mechanism than the observed fast dehydration at 10 m KOH.

The analysis of evolution in Stephens parameters (Figure S3) shows a trend of increasing (4 0 0) parameter and decreasing (0 0 4) parameter for all reactions. This indicates a broadening of peaks in the (*hk*0) family and a narrowing of peaks in the (00*l*) family. This means that there is an increasing crystal size anisotropy over time. Comparing the absolute values of (4 0 0) with (0 0 4) parameters is challenging however as the scales are very different. Interestingly, there appears to be little difference in final values of the (4 0 0) parameters between h‐YMnO_3_ formed in KOH under different temperature and concentration, however, in NaOH, the (4 0 0) parameter appears to be much larger; 670 for 5 m KOH at 320 °C, and 1460 for 5 m NaOH at 320 °C for the final frames. Whereas for the (0 0 4) parameter, high temperature and mineraliser concentration yielded lower values (<10 for 10 m KOH, 320 °C and 5 m KOH, 350 °C and ≈20 for reactions at 300, and 320 °C in 5 m KOH, and 320 °C in 5 m NaOH).

By comparison, many other oxide ferroelectrics, such as BaTiO_3_,^37^, ^38^, ^54^ K_1−*x*_Na_*x*_NbO_3_,^39^, ^40^, ^55^ Sr_1−*x*_Ba_*x*_Nb_2_O_6_,^42^ and BiFeO_3_ 41 the syntheses of which have been previously studied using in situ XRD, do not form via such stable intermediate phases, and as such can be prepared at significantly lower temperatures. These in situ studies show that the outcome and progression of a reaction is highly dependent on the specific system. For example, while BaTiO_3_, Sr_1−*x*_Ba_*x*_Nb_2_O_6_ and KNbO_3_ appear without solid intermediate phases, Sr_1−*x*_Ba_*x*_Nb_2_O_6_ 42 and KNbO_3_ 55 don't appear until after several minutes suggesting the existence of soluble intermediates in the reaction. The reaction producing NaNbO_3_ on the other hand proceeds via several shortly‐lived crystalline intermediates.^55^ Similarly, multiferroic BiFeO_3_ synthesised from nitrates was observed to form highly crystalline Bi_2_O_3_, which remained stable for several minutes before BiFeO_3_ began to crystallise, during this period the Fe(NO_3_)_3_ remained solubilised.^41^

There are still challenges to be addressed with respect to hydrothermal synthesis of h‐YMnO_3_, most notably the difficulty in synthesising phase pure h‐YMnO_3_. This is a result of the complexity of the system, with a total of eight crystalline phases having been detected throughout the course of the reaction. We suggest three possible optimization possibilities for reducing the formation of secondary phases during hydrothermal synthesis; 1) improved mixing of the precursor materials to reduce the diffusion distance between reactive species, thereby reducing the time to form secondary phases, and the use of nanoparticle precursors to increase solubility and reduce solid state diffusion distances, 2) adding Mn^II^ salt to the reaction and performing the reaction under yttrium rich conditions (then dissolving the yttrium hydroxide secondary phase in dilute acid) to reduce the propensity for YMn_2_O_5_ formation or to facilitate its reduction to h‐YMnO_3_ if it does form, and 3) using a lower pressure to reduce the o‐YMnO_3_ formation. Performing the reaction under supercritical conditions (>374 °C, >221 bar) may also provide a route to improving the phase purity, as Nørby et al. showed with yttrium aluminium garnet.^56^

The synthesis procedure used here can be extended to other LnMnO_3_ materials as they are able to be synthesised under similar conditions.^24^ The stability of Ln(OH)_3_ is dependent on the Ln radius, with smaller radii yielding lower stability due to steric effects (Ln is 9‐coordinated in Ln(OH)_3_ and 7‐coordinated in LnO(OH)) and increased acidity of the smaller Ln ions,^24^, ^43^, ^57^ and so a larger range of temperatures are available for studying other systems.

Still, hydrothermal synthesis offers a fast, low‐temperature method for producing h‐YMnO_3_ particles in a range of sizes and shapes which is useful for, for example, catalytic applications, with all reactions reported here taking less than 1 hour. By contrast solid‐state methods for producing powder samples, are only able to produce small (≈100 nm) roughly spherical particles and require much higher temperatures. Thin films are typically required for electronic device application, and the hydrothermal method has been shown to be amenable to deposition on a substrate to make polycrystalline thin films,^58^, ^59^ which could make use of the ferroic properties of h‐YMnO_3_.

## Conclusions

In conclusion, we have studied the hydrothermal synthesis of h‐YMnO_3_ from Y_2_O_3_ and Mn_2_O_3_ as precursors with hydroxide mineraliser, probed through in situ XRD for the first time. The reaction is shown to go via an intermediate period where Y_2_O_3_ is rapidly converted to Y(OH)_3_, which is then dehydrated into YO(OH) before reacting with Mn_2_O_3_ to form h‐YMnO_3_. It is not clear whether dehydration of Y(OH)_3_ is necessary for the reaction to occur; in the reaction performed in NaOH no YO(OH) is observed which suggests that it is not necessary. From extracted kinetic data as a function of temperature, activation energies for the reaction were estimated to be 240±40 and 270±30 kJ mol^−1^ for the reaction in 5 and 10 m KOH, respectively, in line with the high‐temperature required for the reaction to proceed due to the high stability of Y(OH)_3_.

## Experimental Section

Y_2_O_3_ (99.9 %, Alfa Aesar), Mn_2_O_3_ (99.9 %, Sigma Aldrich), KOH (90 %, Sigma Aldrich), NaOH (97 %, Sigma Aldrich) were used for the syntheses.

In situ XRD experiments were conducted at BM01, Swiss Norwegian beamlines (SNBL) at the European Synchrotron Radiation Facility (ESRF). The temperature inside the capillary was calibrated using a hexagonal boron nitride standard prior to experiments. The wavelength of the monochromatic incident beam was 0.77445 Å, refined on a NIST‐660a LaB_6_ reference. The diffracted beam was detected using a 2D Pilatus 2m detector with acquisition times of 2 or 5 s, and processed to 1D diffraction patterns using software available on the PILATUS@SNBL platform.^60^

Samples were prepared for the in situ capillary cell^40^ by weighing out Mn_2_O_3_ and Y_2_O_3_ in equal molar ratio into plastic vials, to which 2 or 5 mL KOH or NaOH solution at 1, 5 or 10 m was added, such that the concentration of Mn ions was 0.347 M. A mass ratio of 85.4:14.6 Mn_2_O_3_:Mn_3_O_4_ was used for the reaction in which Mn_2_O_3_ was partly substituted for Mn_3_O_4_, giving a 95:5 molar ratio of Mn^III^:Mn^II^. The reaction of Y_2_O_3_ with KOH used 0.347 m of Y ions with 5 m KOH.

Reaction mixtures were loaded into an open cell using a syringe, the cell was then sealed with a Swagelok cap screwed on at the end. For a reaction, the cell was pressurised using a Shimadzu LC‐10ADvp HPLC pump to a pressure of 200 bar. This was then heated using a pre‐set hot‐air blower which was moved into place with a motor. The in situ cell made use of a sapphire capillary which is resistant to strongly basic conditions such as those used in this and our previous studies. Stainless‐steel Swagelok parts and tubing were used to form connections between the HPLC pump and the capillary and to seal the reaction cell.^40^

High‐temperature (>250 °C) ex situ reactions were conducted in a 100 mL Monel (copper‐nickel alloy) autoclave. h‐YMnO_3_ was synthesised using a method similar to that described by Stampler et al.^24^ Y_2_O_3_ and Mn_2_O_3_ (total 3 g) were added to the autoclave, to this, 30 mL of 5 m KOH was added, stirred for 30 min, and then heated to 320 °C. It took approximately 40 min to reach the set temperature, the pressure at the set temperature was 90 and 92 bar in the stoichiometric and non‐stoichiometric experiments, respectively. The total reaction time was 6 h (including time to reach the set temperature), after which the autoclave vessel slowly cooled to room temperature over a few hours. The product was collected by vacuum filtration, washed with ≈200 mL of deionised water and dried at 70 °C for 3 h.

All Rietveld refinements were performed using Topas 5.^61^ Batch refinements of synchrotron in situ experiments were run using Topas from launch mode using JEdit with macros for interaction with Topas,^62^ via a custom script written using Jupyter Notebooks^63^ by our group.^55^ Instrumental parameters were determined using a LaB_6_ standard, from which the peak profile was fitted with a Thompson—Cox–Hastings pseudo‐Voigt (TCHZ) peak type.^64^ Crystallographic information files used in refinements were obtained from the Inorganic Crystal Structure Database provided from the following references: h‐YMnO_3_,^2^ o‐YMnO_3_,^65^ Y(OH)_3_,^66^ YO(OH),^67^ YMn_2_O_5_,^68^ Mn_3_O_4_,^69^ Y_2_O_3_,^70^ and Mn_2_O_3_.^71^

Fits for kinetic data were calculated using the SciPy module in Python.^72^ Scale factors were normalised using the highest scale factor value in a dataset, or if there were large fluctuations in the value, the mean of the final values was taken.

Refinements of the diffractograms from the ex situ experiments were modelled by adjusting the scale factor and lattice parameters. This was followed by use of the March–Dollase model for preferred orientation in the 00*l* direction, then in the 1 1 0 direction.^73^ The application of Stephens model for the hexagonal crystal system followed to account for anisotropic crystal size peak broadening.^44^ *B*_iso_ values were then refined, and finally atomic positions. In‐house diffractograms were measured using a Bruker D8 A25 DaVinci X‐ray Diffractometer with Cu_Kα_ radiation, a LynxEye SuperSpeed detector, and a v6 variable incident slit.

## Conflict of interest

The authors declare no conflict of interest.

## Supporting information

As a service to our authors and readers, this journal provides supporting information supplied by the authors. Such materials are peer reviewed and may be re‐organized for online delivery, but are not copy‐edited or typeset. Technical support issues arising from supporting information (other than missing files) should be addressed to the authors.

Supplementary
