# Supplementary material for: A Fast, Low‐Temperature Synthesis Method for Hexagonal YMnO3: Kinetics, Purity, Size and Shape as Studied by In Situ X‐ray Diffraction
Source: Chemistry. 2020 May 12;26(42):9330–7. doi: 10.1002/chem.202000528 (PMC7497076; doi:10.1002/chem.202000528)
Supplement: Supplementary file 1 — Supplementary [file CHEM-26-9330-s001.pdf]

Chemistry–A European Journal

Supporting Information

A Fast, Low-Temperature Synthesis Method for Hexagonal $YMnO_3$: Kinetics, Purity, Size and Shape as Studied by In Situ X-ray Diffraction

Kenneth P. Marshall,[a] Anders B. Blichfeld,[a] Susanne L. Skjærvø,[a] Ola G. Grendal,[a] Wouter van Beek,[b] Sverre M. Selbach,[a] Tor Grande,[a] and Mari-Ann Einarsrud*[a]

Supporting Information

This supporting information contains diffraction patterns of *ex situ* experiments, information on a number of *in situ* experiments performed at the same beam time as those in the main text, and tables with information pertaining to *in situ* and *ex situ* data.

Table S1: Weight fractions at the end of the reactions between Y_2O_3 and Mn_2O_3 under different conditions. Unless otherwise stated, reactions were performed with a 1:1 molar ratio of Y_2O_3:Mn_2O_3 in 5 M KOH.

Temperature (°C)	$Y(OH)_3$	YO(OH)	h-$YMnO_3$	o-$YMnO_3$	YMn_2O_5	Mn_3O_4	Mn_2O_3
300	4.9	0	70	12	13	1.4	0
320	1.5	0	84	3.2	8.5	2.8	0
320 (repeat)	2.1	1.4	92	2.5	2.3	0	0
320 (1 M KOH)	0	32	26	0	33	9	0
300 (10 M KOH)	0	2.6	83	4.9	10	1.6	0
320 (10 M KOH)	0	7.4	76	1.9	12	2.8	0
350 (10 M KOH)	0	5.8	82	4.1	7.6	0	0
320 (5 M NaOH)	0	0	84	3.9	13	0	0
320 (5% Mn(II))	1.9	1.9	86	2.0	5.1	2.2	1.3
350	0	4.3	81	2.5	9.1	3.0	0

Table S2: Refinement parameters for the h-$YMnO_3$ patterns shown in Figure S4.

Sample	March-Dollase (001)	March-Dollase (110)
Stoichiometric	0.510	1.37
10 % excess Y	0.389	1.27

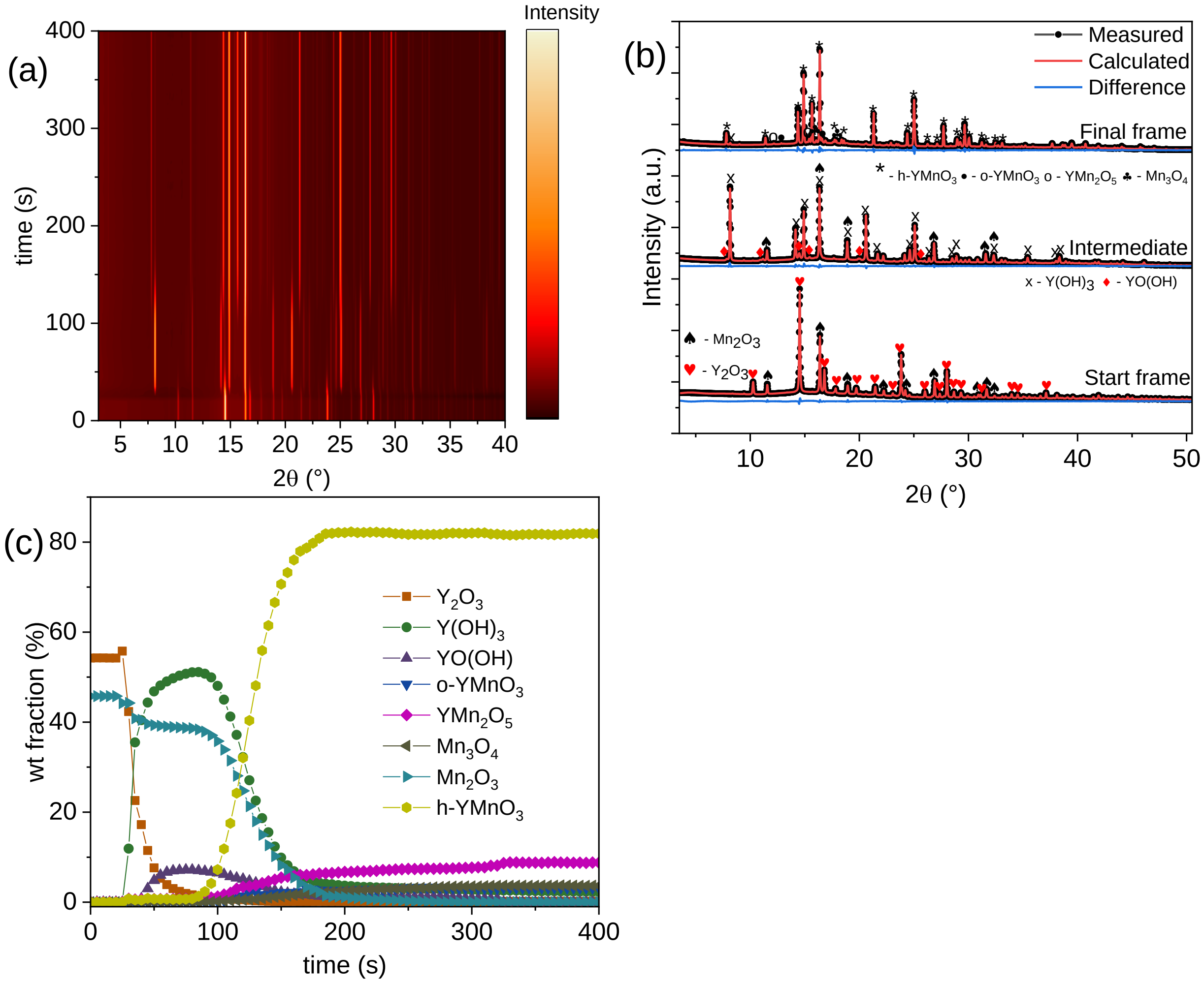

Figure S1: (a) 2D plot of XRD patterns taken over time, **(b)** individual XRD patterns at the beginning, intermediate period and end of the reaction, and **(c)** weight fractions of the phases over time (black vertical line indicates beginning of h-$YMnO_3$ formation) for the reaction between Y_2O_3 and Mn_2O_3 in 5 M KOH at 320 °C.

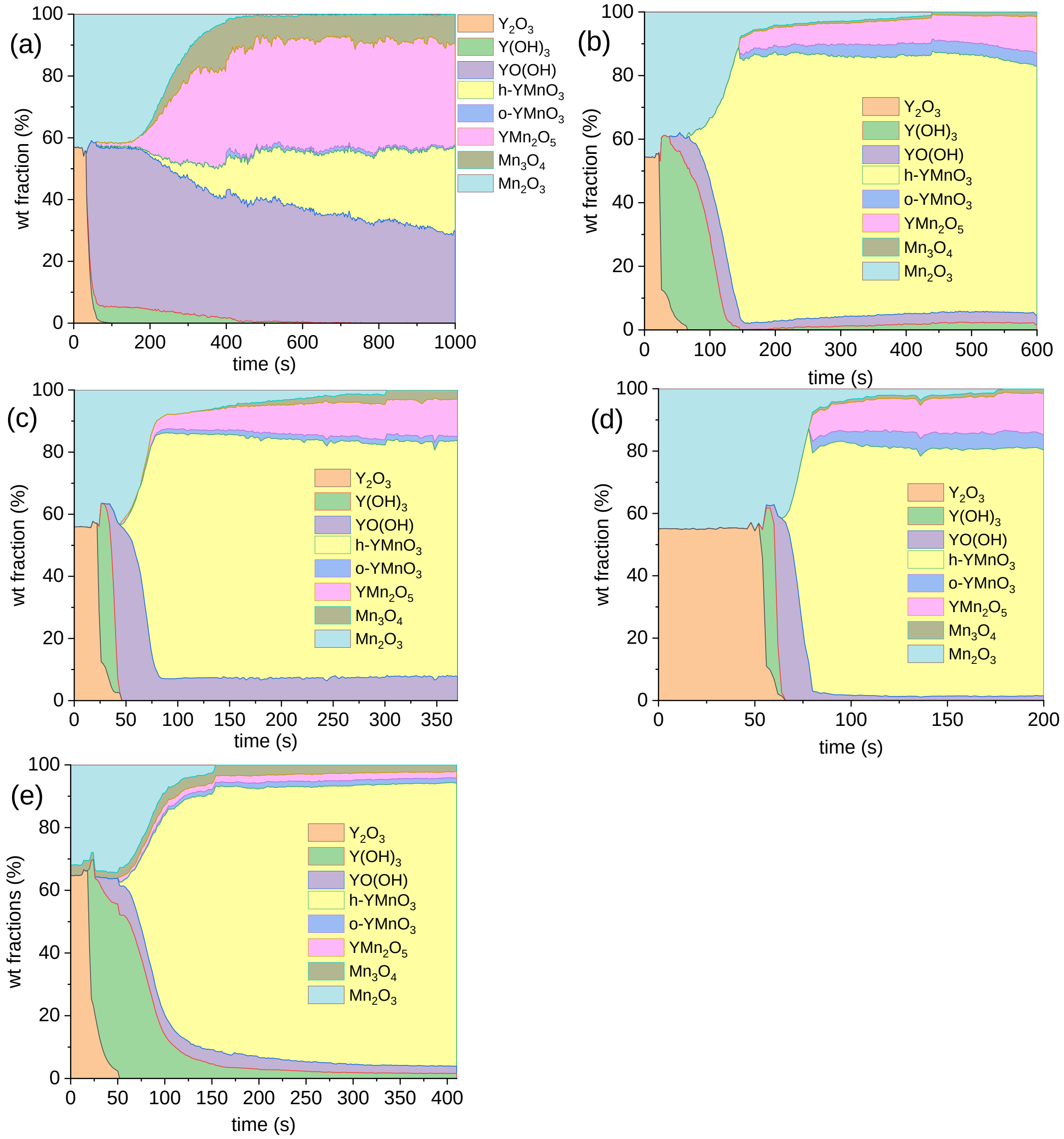

Figure S2: Profile of the reaction of Y_2O_3 and Mn_2O_3 at 320 °C in **(a)** 1 M KOH, 10 M KOH at **(b)** 300 °C (an unidentified phase appeared in this reaction at approximately 200 s, it fits with very highly oriented $Y(OH)_3$, but could only be identified with a single peak with significant overlap with h-$YMnO_3$), **(c)** 320 °C, and **(d)** 350 °C, and **(e)** 5 M KOH with 15 % Mn_2O_3 substituted for Mn_3O_4 such that the ratio of Mn(II) and Mn(III) was 5:95.

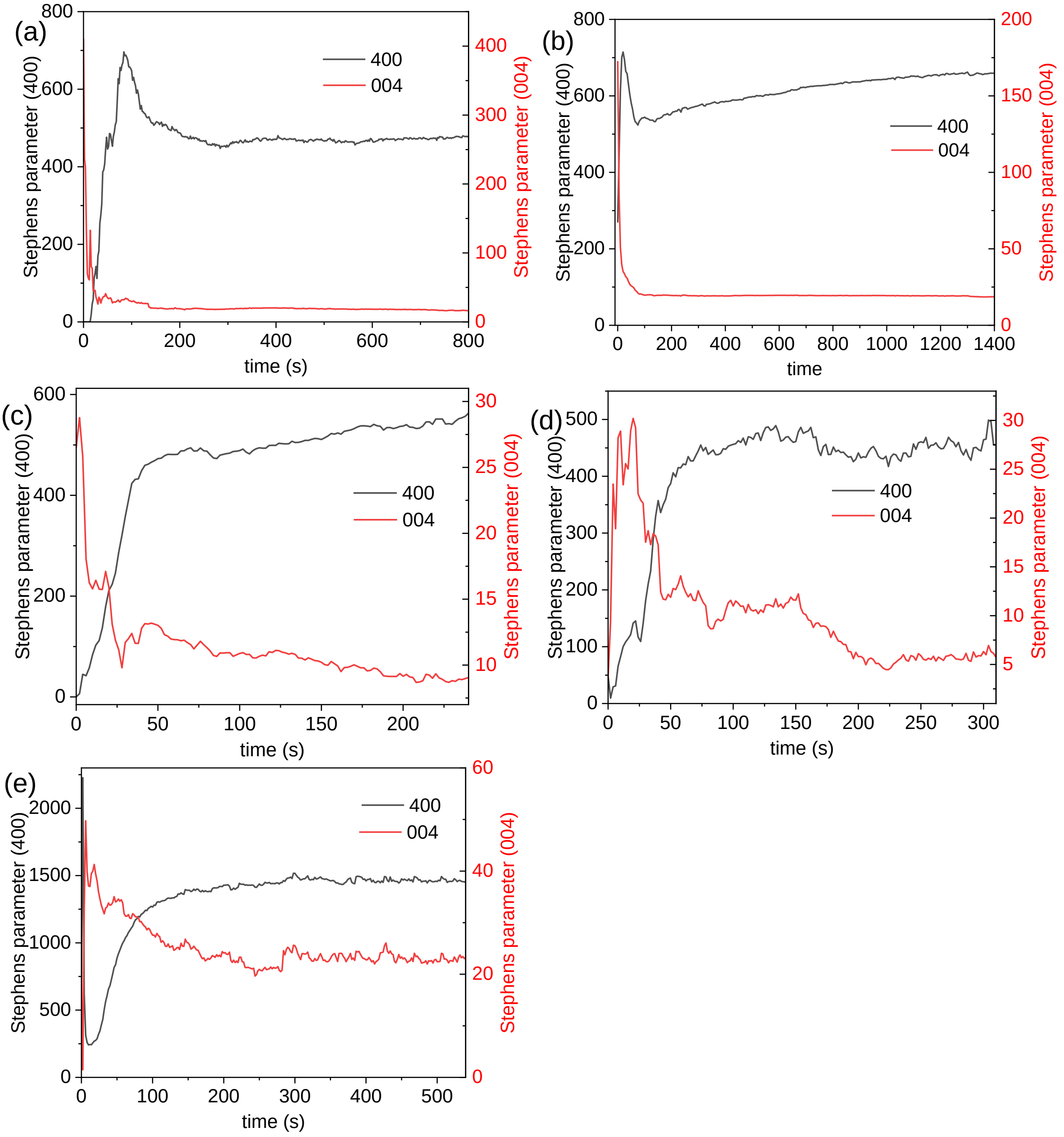

Figure S3: (400) and (004) hexagonal Stephens parameters for h-$YMnO_3$ during formation in 5 M KOH at **(a)** 300 °C, **(b)** 320 °C, and **(c)** 350 °C, **(d)** in 10 M KOH at 320 °C, and **(e)** in 5 M NaOH at 320 °C. Note, the different parameters have different scales, e.g. a larger (400) than (004) parameter does not necessarily mean that peaks in the (*hk*0) family will be broader than those in the (00*l*) family

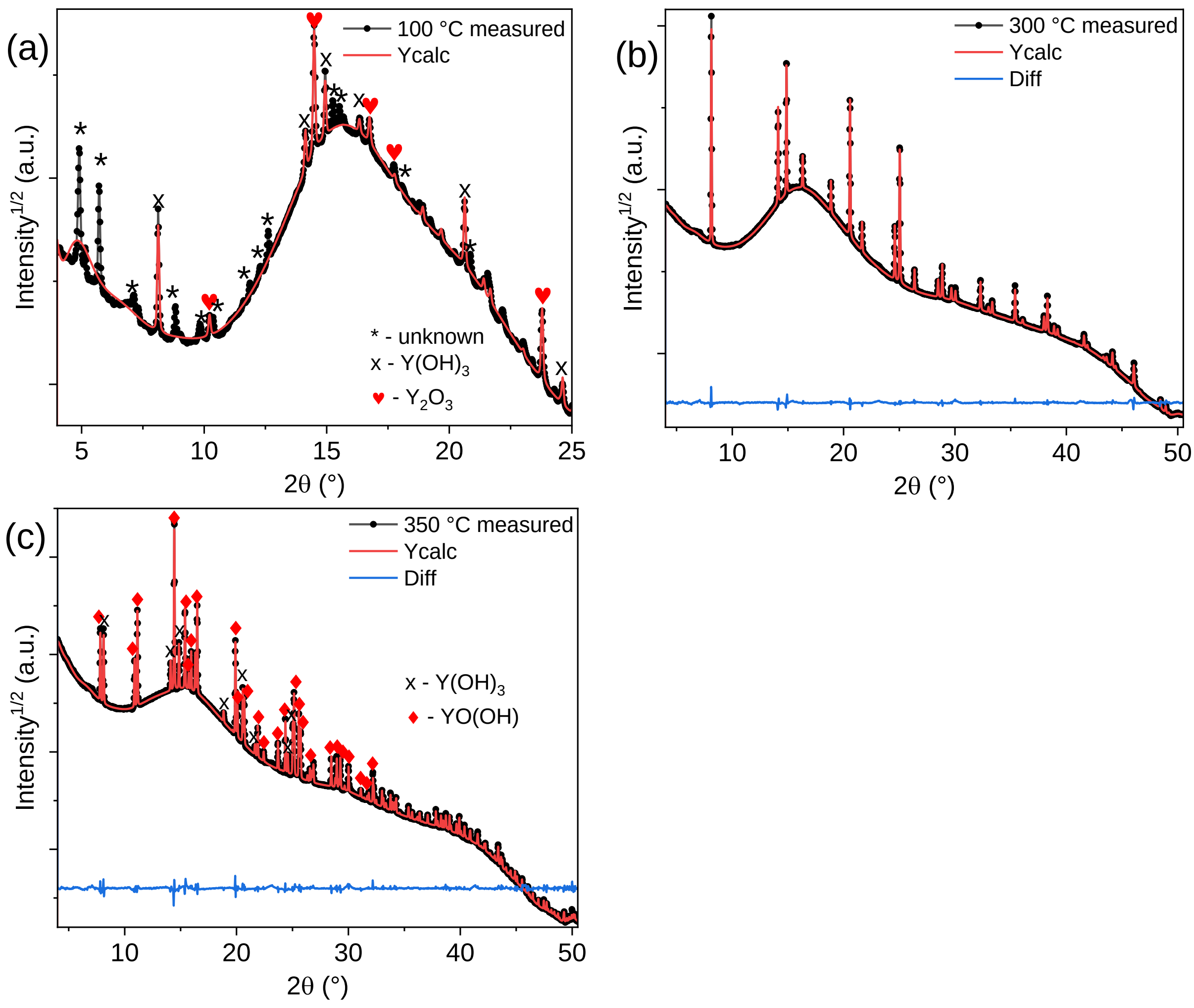

Figure S4: *In situ* XRD patterns of Y_2O_3 in 5 M KOH at **(a)** 100 °C, unidentified diffraction peaks marked with an *, **(b)** 300 °C, and **(c)** 350 °C.

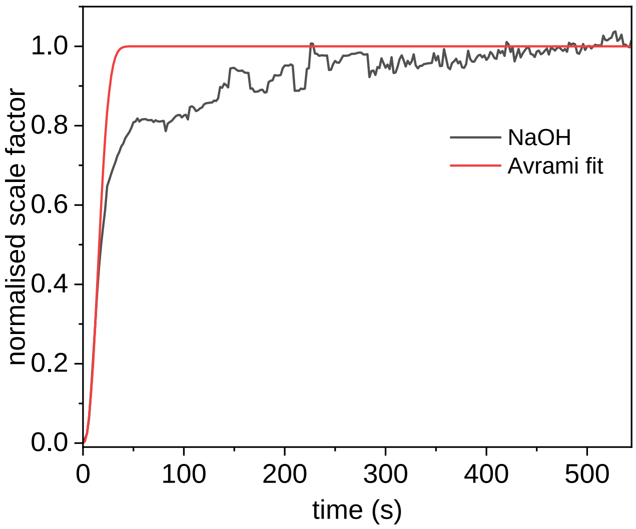

Figure S5: Normalised scale factor of h-$YMnO_3$ over time for the reaction between Y_2O_3 and Mn_2O_3 in 5 M NaOH at 320 °C

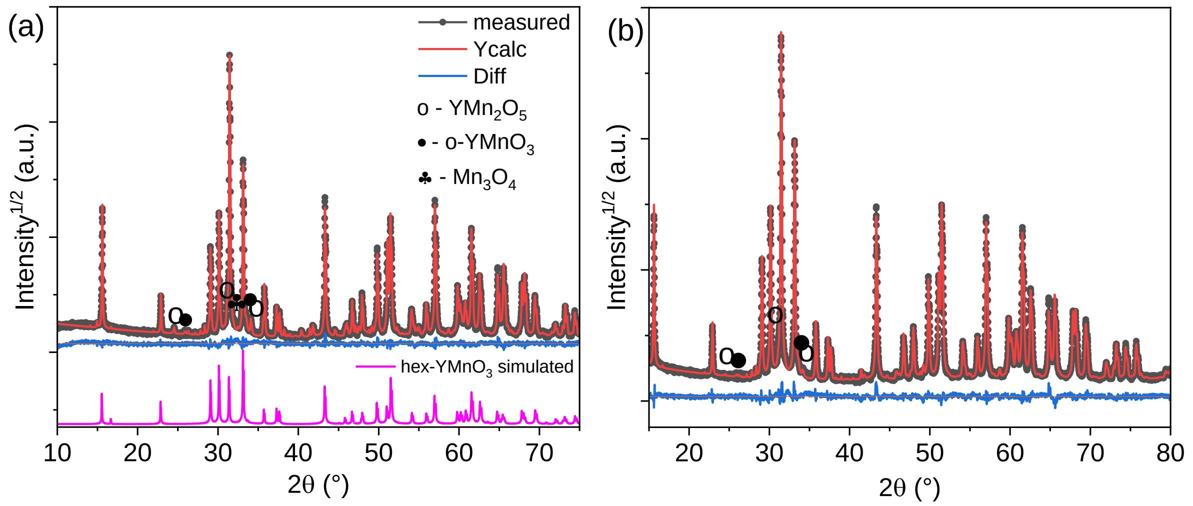

Figure S6: XRD patterns measured *ex situ* (black with circles) with simulated patterns (red) calculated based on Rietveld refinements, and the difference between measured and calculated patterns (blue) and line of zero difference (orange) shown underneath for h-$YMnO_3$ synthesised in an autoclave using **(a)** a stoichiometric ratio of Y_2O_3 and Mn_2O_3, with a simulated pattern of non-orientated h-$YMnO_3$ below (magenta), and **(b)** using a 10 % excess of Y_2O_3.
